# Antimicrobial Prospect of Newly Synthesized 1,3-Thiazole Derivatives

**DOI:** 10.3390/molecules16119386

**Published:** 2011-11-09

**Authors:** Bassem Sadek, Moawia Mohammad Al-Tabakha, Khairi Mustafa Salem Fahelelbom

**Affiliations:** 1 Department of Pharmacology and Therapeutics, College of Medicine and Health Sciences, UAE University, Al-Ain, P.O. Box 17666, United Arab Emirates; 2 Department of Pharmaceutical Sciences, College of Pharmacy, Al-Ain University of Science and Technology, P.O. Box 64141, Al-Ain, United Arab Emirates

**Keywords:** synthesis, antimicrobial evaluation, 1,3-thiazoles, benzo[*d*]thiazoles, partition coefficient

## Abstract

A new series of 1,3-thiazole and benzo[*d*]thiazole derivatives **10–15** has been developed, characterized, and evaluated for *in vitro* antimicrobial activity at concentrations of 25–200 μg/mL against Gram+ve organisms such as methicillin-resistant *Staphylococcus aureus* (*MRSA*), Gram–ve organisms such as *Escherichia coli* (*E. coli*), and the fungal strain *Aspergillus niger* (*A. niger*) by the cup plate method. Ofloxacin and ketoconazole (10 μg/mL) were used as reference standards for antibacterial and antifungal activity, respectively. Compounds **11** and **12** showed notable antibacterial and antifungal activities at higher concentrations (125–200 μg/mL), whereas benzo[*d*]thiazole derivatives **13** and **14** were found to display significant antibacterial or antifungal activity (50–75 μg/mL) against the Gram+ve, Gram–ve bacteria, or fungal cells used in the present study. In addition, a correlation between calculated and determined partition coefficient (log P) was established which allows future development of compounds within this series to be carried out based on calculated log P values. Moreover, compounds **13** and **14** show that the optimum logarithm of partition coefficient (log P) should be around 4.

## 1. Introduction

**Figure 1:**
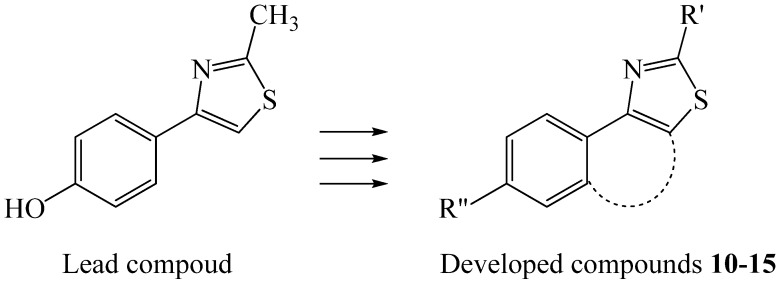
Lead compound 4-(4-hydroxyphenyl)-2-methyl-1,3-thiazole and newly developed compounds **10–15**.

Since discovery and development of effective as well as safe drugs has brought a progressive era in human healthcare that is accompanied by the appearance of drug resistant bacterial strains, there is constant need of new antibacterial agent having novel mechanisms of action to act against the harmful pathogens. The 1,3-thiazole heterocycle is an interesting building block in a variety of natural and synthetic compounds found to possess good antibacterial potential [1,2,3]. Especially, compounds containing the 2-substituted benzo[*d*]thiazole moiety have shown a variety of useful pharmacological actions and many of these have achieved very wide importance in research [4,5,6]. This heterocyclic nucleus is a very important group because of its potent antitumor activity [7,8,9,10] and other significant pharmaceutical utilities, such as treatment of inflammatory diseases, epilepsy, analgesia, viral infections, cancer, and tuberculosis [11,12,13,14,15,16,17]. Encouraged by the above reports and as a part of our ongoing research, the lead compound 4-(4-hydroxyphenyl)-2-methyl-1,3-thiazole has been further developed in the present study to divergent target compounds including substituted 2-phenylbenzo[*d*]thiazole groups and investigated on their antimicrobial activity [18] (Figure 1).

Moreover, several studies were conducted to relate drugs’ lipophilicity, as indicated by their logarithm of partition coefficients, and antimicrobial action [19,20,21]. If such a correlation could be established, this would shorten the time and efforts in synthesizing new chemical entities with improved antimicrobial action. The results published so far regarding this correlation are conflicting. Therefore it was interesting to study the lipophilicity of the prepared homologous compounds in relation to their antimicrobial activity.

## 2. Results and Discussion

### 2.1. Chemistry: General Procedures for Synthesis

The most common and most versatile procedure for the formation of 1,3-thiazoles is the cyclocondensation of a-haloketones with appropriate thioamide derivatives [22,23] In this study, the reaction of commercially available a-bromoketones **1** and **2** with propionthioamide, benzothioamide, and 4-methoxybenzothioamide under basic conditions furnished the compounds **10–12** respectively (Scheme 1). The formation of benzo[*d*]thiazoles **13–15** was achieved through a cyclocondensation reaction of corresponding substituted benzoic acid **3a–c** and 2-aminothiophenol in the presence of a catalytic amount of polyphosphoric acid [8,24]. Finally, proceeding from the different thiazole derivatives **4**–**9**, methyl ether cleavage with BBr_3_ in dichloromethane provided the corresponding phenols **10**–**15** in high yields [25].

**Scheme 2:**
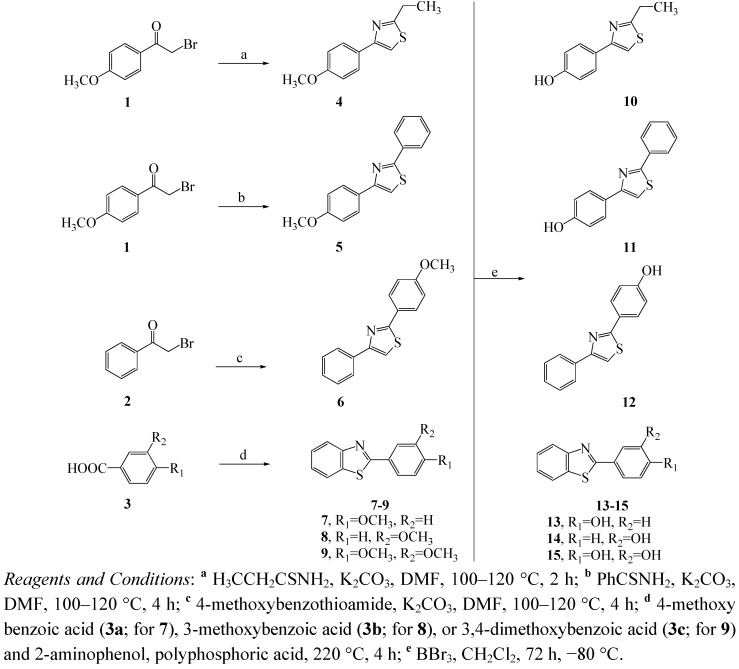
Synthesis of thiazole derivatives **10**–**15**.

### 2.2. Biological Activity

The 2-ethyl-1,3-thiazole derivative **10** with an extended alkyl chain exhibited low antimicrobial activity (MIC 200 μg/mL) compared to the lead compound (MIC 150–200 μg/mL) and the reference substances ofloxacin and ketoconazole, demonstrating that further lengthening of the alkyl moiety at the 2-position and thereby an increase of lipophilicity did not enhance antimicrobial potency. On the other hand, a minor increase in antimicrobial activity was achieved with the next two compounds, the 2-phenyl-1,3-thiazole derivatives **11**–**12**. Among both structural isomers which differ from each other solely in the position of 4-hydroxyphenyl moiety at the heterocycle 1,3-thiazole, compound **12** having a 4-hydroxyphenyl at the 2-position of 1,3-thiazole exhibited a MIC of 125–150 μg/mL against *S. aureus*, *E. coli*, and *A. niger*, whereas **11** having the 4-hydroxyphenyl at the 4-position of 1,3-thiazole exhibited significant lower antimicrobial activity (MIC 150–200 μg/mL) against the tested microorganisms. Major improvement in antimicrobial activity was achieved through structural development of benzo[*d*]thiazole derivatives **13–15**. Compounds **13** and **14** sharing as a common feature the presence of a 4-hydroxyphenyl substituent at the 2-position of the benzo[*d*]thiazole nucleus displayed promising antibacterial activities (MIC 50–75 μg/mL) against all tested culture strains used in the present study, indicating that a structural modification of 1,3-thiazole heterocyle to benzannelated 1,3-thiazole derivatives positively influenced the antimicrobial activity. In contrast, the antimicrobial results observed for compound **15** (MIC 125 μg/mL) was clearly lower, pointing out that a monohydroxysubstitution is most favorable among the newly developed and tested series (Table 1).

**Table 1:** Antimicrobial activity of the title compounds along with the experimental “log P” and the calculated “Clog P”.

		MIC µg/mL			Lipophilicity			
Compound	Structural formula	*S. aureus*	*E. coli*	*A. niger*		Log P	Clog P **	
Lead *	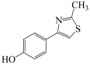	150 *	200 *	150 *		2.68	2.89	
**10**	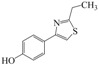	200	200	200		3.41	3.20	
**11**	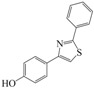	150	200	150		4.65	4.24	
**12**	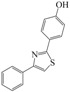	125	150	125		4.45	4.24	
**13**	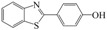	50	50	75		3.86	4.07	
**14**	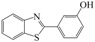	50	75	50		3.83	4.05	
**15**	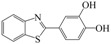	125	125	125		2.98	3.14	
**Ofloxacin**		10	12.5	--		--	--	
**Ketoconazole**		--	--	12.5		--	--	

* Previously published [18]; ** Using ALOGPS 2.1 software

### 2.3. Lipophilicity and QSAR

**Figure 2:**
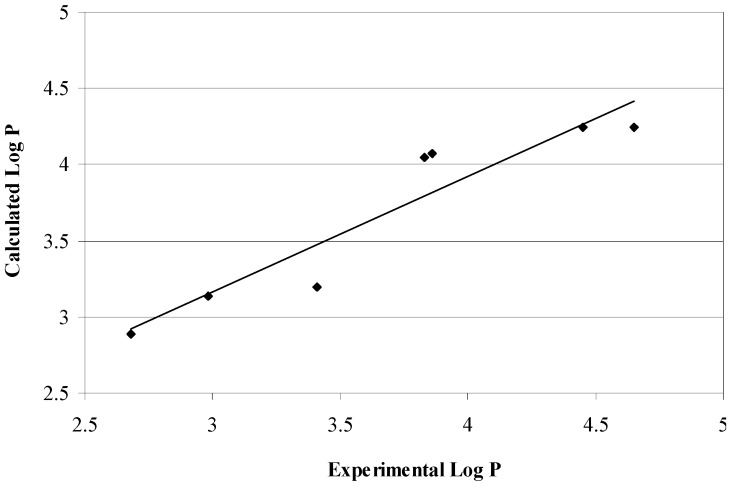
Correlations between the experiemtnal and the calculated log P.

A good correlation was found between the experimentally determined partition coefficients and the predicted ones (r = 0.94), as shown in Figure 2. The high lipophilicity of the synthesized compounds (log P 2.98–4.65) promoted us to investigate the possibility of a correlation between the type of chemical substitution and the antimicrobial activity of the title compounds, therefore, the linear regression of the partition coefficient with the biological activity expressed as MIC (μg/mL) for the synthesized compounds were studied.

**Figure 3:**
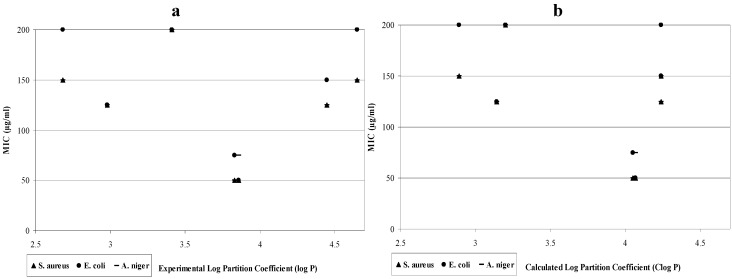
Influence of the prepared compounds lipophilicity determined experimentally “log P” (Figure 3a) and as predicted “Clog P” (Figure 3b).

The results show that correlations between lipophilic properties and MICs against different microbial species are not statistically significant (Figure 3a,b). The results can be explained according to previous studies that attributed poor correlations to the influence from several factors including the fact that not all homologous series will necessarily show correlations [26]. Additionally, the microorganism strains tested and the suggestions that there is an optimum partition coefficient rather than direct correlation may be the reasons for observed results [27]. The current study shows an optimum log partition coefficient around 4, as suggested by compounds **13–14** (calculated and experimentally determined log P is 3.83–4.07).

## 3. Experimental

### 3.1. General

Melting points are uncorrected and were determined in open capillaries in a Buechi 512 Dr. Tottoli apparatus. ^1^H-NMR spectra were recorded on a Bruker WC 300 spectrometer with tetramethylsilane (TMS) as internal standard. Chemical shifts are reported in ppm downfield from internal tetramethylsilane used as reference. ^1^H-NMR signals are reported in order: multiplicity (s, single; d, doublet; t, triplet; q, quantet; m, multiplet; *, exchangeable by D_2_O), number of protons, and approximate coupling constants in Hertz. ^13^C-NMR spectra were recorded on a Bruker DPX 400 Avance (100 MHz) instrument. Chemical shifts are reported in ppm downfield from internal tetramethylsilane used as reference. Elemental analyses were performed on Perkin-Elmer 240B and 240C instruments. Analyses (C, H, N) indicated by the symbols of elements were within ±0.4% of the theoretical values. Chromatographic separations were done using a Chromatotron Model 7924 (Harrison Research) with 4-mm layers of silica gel 60 PF containing gypsum (Merck). EI-mass spectra were recorded using Finnigan MAT CH7A (70 eV), Finnigan MAT 711 (80 eV), or Kratos MS 25 RF (70 eV). ^+^FAB-MS spectra were recorded on Finnigan MAT CH5DF instrument (xenon, DMSO)/ glycerol). The following abbreviations are used: *N*,*N*-Dimethylformamide, DMF; EtOH, ethanol; Et_2_O, diethyl ether; MeOH, methanol; Me_2_SO, dimethyl sulfoxide; Ph, phenyl; Th, thiazole; Benz, benzene; BTH, benzothiazole.

#### 3.1.1. *2-Ethyl-4-(4-methoxyphenyl)thiazole* (**4**)

4-Methoxyphenacyl bromide (**1**, 1.49 g, 6.5 mmol), propionthioamide (0.47 g, 6.5 mmol), and K_2_CO_3_ (1.20 g, 8.7 mmol) were dissolved in dry DFM (30 mL). The mixture was stirred for 4 h at 100–120 °C, cooled, and the solvent was removed under reduced pressure. The solid residue was recrystallized from EtOH. Yield 75%; mp 66–67 °C; ^1^H-NMR δ ppm = 1.34 (t, *J* = 7.52 Hz, 3H, CH_3_), 2.99 (q, *J* = 7.5 Hz, 2H, CH_2_), 3.79 (s, 3H, H_3_CO), 6.99 (d, *J* = 8.7 Hz, 2H, Ph-3,5H), 7.74 (s, 1H, Th-5H), 7.87 (d, *J* = 8.7 Hz, 2H, Ph-2,6H); EI-MS m/z (%) 220 (M^+^, 100).

#### 3.1.2. *4-(4-Methoxyphenyl)-2-phenylthiazole* (**5**)

4-Methoxyphenacyl bromide (**1**, 1.49 g, 6.5 mmol), benzothioamide (0.89 g, 6.5 mmol), and K_2_CO_3_ (1.20 g, 8.7 mmol) were dissolved in dry DMF (30 mL). The mixture was stirred for 2 h at 100–120 °C, cooled, and the solvent was removed under reduced pressure. The solid residue was recrystallized from EtOH. Yield 73%; mp 96.5–98 °C; ^1^H-NMR δ ppm = 3.82 (s, 3H, H_3_CO), 6.68–6.90 (m, 2H), 7.35–7.38 (m, 3H), 7.44–7.48 (m, 2H), 7.84 (s, 1H, Th-5H), 7.86–7.90 (m, 2H); EI-MS m/z (%) 268 (M^+^, 100).

#### 3.1.3. *2-(4-Methoxyphenyl)-4-phenylthiazole* (**6**)

Phenacyl bromide (**2**, 1.49 g, 6.5 mmol), 4-methoxybenzothioamide (1.28 g, 6.5 mmol), and K_2_CO_3_ (1.20 g, 8.7 mmol) were dissolved in dry DMF (30 mL). The mixture was stirred for 2 h at 100–120 °C, cooled, and the solvent was removed under reduced pressure. The solid residue was recrystallized from EtOH. Yield 73%; mp 93.5–95 °C; ^1^H-NMR δ ppm = 3.85 (s, 3H, H_3_CO), 6.93–6.97 (m, 2H), 7.36–7.39 (m, 3H), 7.44–7.74 (m, 2H), 7.85 (s, 1H, Th-5H), 7.88–7.92 (m, 2H); EI-MS m/z (%) 268 (M^+^, 100).

#### 3.1.4. *2-(4-Methoxyphenyl)benzo[d]thiazole* (**7**)

Equimolar amounts of 4-methoxybenzoic acid and *o*-aminothiophenol were added to polyphosphoric acid (15 g) and the mixture was refluxed for 4 h at 220 °C. The reaction mixture was cooled and poured in ice cold 10% sodium carbonate solution. The precipitate was filtered and recrystallised from methanol [8,24]. Yield 90%; mp 101 °C; ^1^H-NMR δ ppm = 3.87 (s, 3H, H_3_CO), 6.81 (d, *J = *8.6 Hz, 2H, Ph-3,5H), 7.55 (d, *J* = 8.6 Hz, 2H, Ph-2,6H), 7.5–8.2 (4H, m, 2-BTH); EI-MS m/z (%) 242 (M^+^, 80). Anal. (C_14_H_11_NOS): C, H, N Calcd. 69.68, 4.59, 5.80; Found. 69.89, 4.85, 5.25. 

#### 3.1.5. *2-(3-Methoxyphenyl)benzo[d]thiazole* (**8**)

Yield 84%; mp 98–99 °C; ^1^H-NMR δ ppm = 3. 81 (s, 3H, H_3_CO), 6.5–7.2 (4H, m, Ph-H), 7.5–8.2 (4H, m, 2-BTH); EI-MS m/z (%) 242 (M^+^, 100). Anal. (C_14_H_11_NOS): C, H, N Calcd. 69.68, 4.59, N, 5.80; Found. 69.75, 4.78, 5.44. 

#### 3.1.6. *2-(3,4-Dimethoxyphenyl)benzo[d]thiazole* (**9**)

Yield 81%; mp 103–105 °C; ^1^H-NMR δ ppm = 3.79 (s, 6H, 2 H_3_CO), 6.6–7.2 (3H, m, Ph- H), 7.5–8.2 (4H, m, 2-BTH); EI-MS m/z (%) 272 (M^+^, 100). Anal. (C_13_H_9_NO_2_S): C, H, N Calcd. 64.18, 3.73, 5.76; Found. 64.25, 3.81, 5.80. 

### 3.2. General Procedure for Ether Cleavage

A solution of the corresponding ether (4 mmol) in dry CH_2_Cl_2_ (20 mL) was cooled to −80 °C under exceeding −60 °C. The reaction mixture was then allowed to warm to room temperature and stirred for additional 72 h. Subsequently, the mixture was cooled to −80 °C and MeOH (25 mL) was added dropwise. The organic layer was removed from the mixture under reduced pressure. After addition of a saturated K_2_CO_3_ solution in H_2_O to the aqueous layer, the crude product precipitated. It was isolated by filtration and recrystallized from EtOH.

#### 3.2.1. *2-Ethyl-4-(4-hydroxyphenyl)thiazole* (**10**)

From **4**. Yield 70%; mp 155 °C; ^1^H-NMR δ ppm = 1.34 (t, *J* = 7.52 Hz, 3H, CH_3_), 2.99 (q, *J* = 7.5 Hz, 2H, CH_2_), 6.81 (d, *J* = 8.6 Hz, 2H, Ph-3,5H), 7.55 (d, *J* = 8.6 Hz, 2H, Ph-2,6H), 7.74 (s, 1H, Th-5H), 10.45 (s*, 1H, OH); ^13^C-NMR (CDCl_3_) δ ppm = 161.71 (Ph-1C), 128.70 (Ph-3,5C), 120.40 (Ph-4C), 114.70 (Ph-2,6C), 111.89 (Th-CH), 42.34 (CH_2_), 22.17 (CH_3_); EI-MS m/z (%) 206 (M^+^, 100). Anal. (C_11_H_11_NOS): C, H, N Calcd. 64.36, 5.40, 6.82; Found. 64.16, 5.52, 6.98.

#### 3.2.2. *4-(4-Hydroxyphenyl)-2-phenylthiazole* (**11**)

From **5**. Yield 72%; mp 159 °C; ^1^H-NMR δ ppm = 6.68–6.90 (m, 2H), 7.34–7.37 (m, 3H), 7.45–7.49 (m, 2H), 7.85 (s, 1H, Th-5H), 7.87–7.91 (m, 2H), 10.43 (s*, 1H, OH); ^13^C-NMR (CDCl_3_) δ ppm = 166.53 (quat), 159.65 (quat), 139.09 (quat), 138.05 (CH), 133.62 (quat), 129.70 (CH), 128.95 (2CH), 128.82 (2CH), 127.84 (2CH), 126.13 (quat), 114.40 (2CH); EI-MS m/z (%) 254 (M^+^, 100). Anal. (C_15_H_11_NOS): C, H, N Calcd. 71.12, 4.38, 5.53; Found. 71.01, 4.49, 5.49.

#### 3.2.3. *2-(4-Hydroxyphenyl)-4-phenylthiazole* (**12**)

From **6**. Yield 72%; mp 123 °C; ^1^H-NMR δ ppm = 6.93–6.97 (m, 2H), 7.36–7.39 (m, 3H), 7.44–7.74 (m, 2H), 7.85 (s, 1H, Th-5H), 7.88–7.92 (m, 2H), 9.98 (s*, 1H, OH); ^13^C-NMR (CDCl_3_) δ ppm = 168.28 (quat), 161.05 (quat), 143.34 (quat), 139.09 (quat), 138.05 (quat), 129.70 (CH), 128.00 (2CH), 127.84 (2CH), 126.55 (quat), 117.83 (CH), 114.25 (2CH); EI-MS m/z (%) 254 (M^+^, 100). Anal. (C_15_H_11_NOS): C, H, N Calcd. 71.12, 4.38, 5.53; Found. 71.31, 4.31, 5.48.

#### 3.2.4. *2-(4-Hydroxyphenyl)benzo[d]thiazole* (**13**)

From **7**. Yield 87%; mp 169–170 °C; ^1^H-NMR δ ppm = 6.81 (d, *J = *8.6 Hz, 2H, Ph-3,5H), 7.55 (d, *J* = 8.6 Hz, 2H, Ph-2,6H), 7.5–8.2 (4H, m, 2-BTH), 10.12 (s*, 1H, OH); ^13^C-NMR (CDCl_3_) δ ppm = 167.96 (Th-2C), 158.50, 154.05 (Th-4C), 134.97 (Th-5C), 133.52, 128.72 (Ph-3,5C), 128.32 (CH), 124.82 (CH), 121.81 (2CH), 114.46 (Ph-2,6C); EI-MS m/z (%) 228 (M^+^, 100). Anal. (C_13_H_9_NOS): C, H, N Calcd. 68.70, 3.99, 6.16; Found. 69.01, 4.21, 6.24.

#### 3.2.5. *2-(3-Hydroxyphenyl)benzo[d]thiazole* (**14**)

From **8**. Yield 81%; mp 159–161 °C; ^1^H-NMR δ ppm = 6.81 (d, *J = *8.6 Hz, 2H, Ph-3,5H), 7.55 (d, *J* = 8.6 Hz, 2H, Ph-2,6H), 7.5–8.2 (4H, m, 2-BTH), 9.98 (s*, 1H, OH); EI-MS m/z (%) 228 (M^+^, 100). Anal. (C_13_H_9_NOS): C, H, N Calcd. 68.70, 3.99, 6.16; Found. 68.92, 4.12, 6.28.

#### 3.2.6. *2-(3,4-Dihydroxyphenyl)benzo[d]thiazole* (**15**)

From **9**. Yield 63%; mp 154–156 °C; ^1^H-NMR δ ppm = 6.6–7.2 (3H, m, Ph- H), 7.5–8.2 (4H, m, 2-BTH), 8.91 (1H, br s, Ph-OH), 9.08 (1H, br s, Ph-OH); EI-MS m/z (%) 244 (M^+^, 100). Anal. (C_13_H_9_NO_2_S): C, H, N Calcd. 64.18, 3.73, 5.76; Found. 64.25, 3.81, 5.80. 

### 3.3. Partition Coffeicient Determination

The log partition coefficients of the compounds were determined experimentally (log P) and were also calculated using ALOGPS 2.1 software (Clog P). The experimental log P was determined using the classical shake-flask method employing 1-octanol and aqueous phosphate buffer (pH 7.2). Both experimentally determined and calculated partition coefficients were examined for their correlation with each other and with the observed minimum inhibitory concentrations. 

### 3.4. Antimicrobial Activity

The quantitative *in vitro* antimicrobial study was carried on Muller-Hinton agar (Hi-media) plates (37 °C, 24 h) by the agar diffusion cup plate method [28]. The compounds (200–25 μg/mL) were screened for antimicrobial activity against the bacterial strains *Staphylococcus aureus* ATCC 25923 (*S. aureus*) (Gram+ve) and *Escherchia coli* ATCC 35218 (*E. coli*) (Gram-ve). Antifungal activity was tested on Sabouraud dextrose agar (Hi-media) plates (26 °C, 48–72 h) by the cup plate method against *Aspergillus niger* A733 (*A. niger*) also at a concentration level of 200–25 μg/mL. Ofloxacin and ketoconazole were used as standards for comparison of antibacterial and antifungal activity under the similar conditions. DMF was used as a solvent control for both antibacterial and antifungal activities, and the results are presented in minimal inhibition concentration (MIC) values (μg/mL). 

## 4. Conclusions

The new compounds **10**–**16** presented here obviously differ in their corresponding antimicrobial activity depending on the type of the heterocycle and the position of substituents. In the course of this study, derivative **12** possessing a 4-hydroxyphenyl group at the 2-position of the 1,3-thiazole moiety in particular was identified as showing moderately enhanced antibacterial activity against methicillin-resistant *S. aureus *(Gram positive) and *E. coli *(Gram negative) bacteria and antifungal activity against *A. niger*, as compared to its structural isomer **11**. Moreover, structural development of benzannelated 1,3-thiazoles led to benzo[*d*]thiazole derivatives **14** and **15** with significantly improved *in vitro* antibacterial activity. A relationship could not be established between the compounds partition coefficient and the antibacterial activity, although an optimum log P of approximately 4 was observed which could be used to generate further structural optimization among this series. These results, combined with the potential benefits or at least differences in pharmacokinetics make the title 1,3-thiazole and benzo[*d*]thiazole classes interesting leads for future study of their mechanism of action and detailed structure-activity relationship studies. One can conclude that a comprehensive study by using new potent compounds of this series should be conducted in order to obtain more significant correlations between the chemical structures of the compounds and their antimicrobial activity. 
